# Medical History from the Earliest Times

**Published:** 1893-04-01

**Authors:** E. T. Withington


### Medical History from the Earliest Times.

By E. T. Withington, M.A., M.B. Oxon.

*(Continued from page 389, Vol. XIII.)*

### XLVIII.—PARACELSUS (*continued*).

According to Paracelsus the physicians of his day wasted their time disputing with peasants and laymen, and neglected "philosophy," which would have taught them the doctrine of "tartar," and its coagulation by the "spirit of salt," "a far better anatomy than the cutting up of robbers and such like, for the young new-fledged fools when they have seen it all know less than before, and choke themselves in dirt and carcases." All food contains poisonous, as well as nutritious ingredients, and it is the business of the Alchemist or Archeus of digestion to separate the two. If he fails to do this, or if the excretory organs are damaged, the poison remains in the body, and there meets with a "spirit of coagulation," which solidifies and causes it to be deposited on the teeth, in the joints, and in various other organs. This deposit Paiacelus calls "tartar," partly because of its resemblance to that found in wine casks, and partly because the pains it causes are like those of Tartarus. Numerous "tartaric diseases" are thus produced, the chief being gout and stone, which are closely allied, and the tendency to which may be inherited. This doctrine of tartar is one of the most highly-praised parts of the Paracelsic system, but though it contains much that is true there is little that is new, as Paracelsus might have learnt had he read his Galen instead of burning him.

Still more excellent is his teaching as to the healing of wounds. Every part of the body, he says, contains a natural "balsam" capable of curing injuries, and nothing more is required than some simple ointment to serve as a nutriment to this balsam, and dressings to protect it from "elementary powers hostile to nature." Here, one might say, is the Hunterian doctrine of plastic lymph, and the modern theory of antisepsis in a single sentence. The hostile powers of the air may indeed be considered a poetic anticipation of micro-organisms, but the rest of the sentence is to be found in, and was probably copied from, the writings of Basil Valentine.

In Paracelsus the belief in the healing power of nature is overshadowed by his faith in the efficacy of drugs. Every disease has its specific remedy or "arcanum," and is only incurable because of the ignorance of physicians. Those who say that leprosy is incurable are not only ignorant but impious, for did not Christ specially command His disciples to heal lepers? He applies the same principles to surgery, "which requires more physic than all internal diseases." Operations, with the possible exception of lithotomy are barbarous, and it should be the surgeon's great object to discover the infallible arcana which cure all diseases, surgical as well as medical. It is thus that Paracelsus arrives at his highly praised, though by no means original assertion that surgery and medicine are one, but it is obvious that, had such doctrines been accepted, they would have reduced the former art to a mere caricature of the latter.

Magnetism has always been an attractive subject for the mystics, and the power of the load-stone, "which traverses many thousand miles to its polar star," is adduced by Basil Valentine to prove that the curative influence of sympathetic ointments applied to the blood which has flowed from, or the weapon which has inflicted, a wound is carried to it through the air, and will undoubtedly heal it. Paracelsus repeats this, and adds a discovery of his own, viz., that magnets properly applied will check all abnormal discharges, bleeding, &c., and will also cure epilepsy and tetanus, for which purpose four magnets must be fastened to the four extremities, and then the proper medicines be given. "And this paragraph is worth more than all the humoralists have written or taught in all the schools from their first day until now."

In the choice of drugs Paracelsus relies mainly on the doctrine of signatures: "As a woman is known by her shape, so the medicines," and the nature of the arcanum is recognised by the form, and especially by the colour, "tincture," of the substances containing it. Thus the topaz and the juice of the celandine are useful in jaundice, but yellow substances are also beneficial in heart disease, yellow being the colour of the sun, which rules that organ, and to distinguish which is adapted to which the physician must acquire the knowledge of philosophy, astronomy, and alchemy above mentioned. In one passage Paracelsus asserts that the action of a remedy depends not on its amount, but on the virtue of the arcanum, which he compares to a spark, but elsewhere he says that the dose must be regulated by the quantity (copia) of the disease, for when these are equivalent "nature at once cures that which is contrary to it."

Simplicity in prescribing is the natural result of the doctrine of specifics, and Paracelsus' attack on the complicated mixtures of his contemporaries is one of his best claims to the title of reformer. "Which are the best breeches? Those which are whole; the patched are the worst." His claims to be the introducer of new, and particularly of chemical remedies, are very questionable. The recommendation of antimony, round which the contests between the Galenists and "Chemists" specially centred, has been shown to be taken almost verbatim from Basil Valentine. Mercury and opium were used long before his time, and there is no reason to believe that the arcanum which Paracelsus called "laudanum" had anything to do with the preparation so named by Sydenham. But if it may be questioned whether Paracelsus disproved any part of the Galenic system, or introduced a single new truth into medicine, the controversies which raged round his doctrines doubtless assisted in overthrowing the old theories; and though his attack consisted chiefly in noisy declamation, with occasional happy hits, it is these which most influence the vulgar, among whom discarded medical theories tend to linger longest. The walls of the Galenic Jericho were tottering with age, and were already undermined by the knife of the anatomist, but it may also have been necessary that someone should blow the trumpet, and we may admit that Paracelsus blew vigorously.

More space has been devoted to Paracelsus than he probably deserves, and the judgment has been an unfavourable one; but if some things are omitted which are to his credit, we have passed over far more of his absurdities. His admirers praise his wonderful strength of mind in breaking with the old traditions. It may, indeed, excite our admiration when a man like Vesalius stakes his reputation and livelihood on the truth of facts denied by nearly all his fellows, and triumphantly demonstrates them; but what sort of mind is required for the assertion that all one's predecessors were consummate idiots, and for the declamation of mystical theories as incapable of disproof as the existence of ghosts or Mahatmas, may be left to the admirers of Paracelsus to determine. There can be no temptation to judge harshly now one who was harshly judged in his lifetime, but when the notorious German is placed above Harvey and Vesalius, and by the side of Hippocrates, we may at least attempt to dislodge him, and to reduce him to his proper level—not, indeed, that of a mere drunken quack, but below Harvey, below Vesalius, and below the man whom he robbed in the dark, the unknown monk of Erfurt who called himself Basil Valentine.

